# Case Report: Spinal cord stimulation for phantom limb pain facilitates upper limb myoelectric prosthesis use

**DOI:** 10.3389/fpain.2026.1751694

**Published:** 2026-03-26

**Authors:** Lauren E. Penz, Andrew W. Nelson, F. Clay Smither, Jonathan M. Hagedorn

**Affiliations:** 1 Division of Physical Medicine and Rehabilitation, Mayo Clinic, Rochester, MN, United States; 2 Limb Lab, Rochester, MN, United States; 3 Division of Pain Medicine, Mayo Clinic, Rochester, MN, United States

**Keywords:** amputee, case report, myoelectric prosthesis, phantom limb pain, spinal cord stimulation

## Abstract

**Introduction:**

This case describes the application of spinal cord stimulation for the treatment of phantom limb pain in a patient with a left transhumeral amputation. Treatment of pain involving a high frequency spinal cord stimulator allowed the patient to utilize his myoelectric prosthesis and improve his overall function.

**Clinical findings:**

Severe, phantom limb pain with associated involuntary muscle contractions prevented the patient from wearing a prosthesis for more than three hours per day. He also had difficulty using his myoelectric prosthesis to use his left upper extremity for functional tasks.

**Therapeutic intervention:**

A high frequency spinal cord stimulator was placed at the level of the cervical spine. The patient had improvement in pain and overall function. He was also able to tolerate wearing his prosthesis longer each day.

**Conclusion:**

High frequency spinal cord stimulation may be an effective treatment option for individuals with phantom limb pain. Treatment of phantom limb pain may decrease barriers to prosthesis use and improve overall function.

## Introduction

It is estimated that by the year 2050, 3.6 million people will be living with the loss of a limb (1). The prevalence of phantom limb pain (PLP) is approximately 64% (2). PLP, like other chronic pain conditions, may disrupt function due to pain intensity and frequency. Post-amputation pain conditions may also be a barrier to prosthesis use which may prevent functional use of the affected limb.

Current treatment options for PLP are numerous and include medications such as gabapentinoids, tricyclic antidepressants, opioids, and sodium channel blockers; physical therapy; occupational therapy including mirror therapy; nerve blocks; neuromodulation; and surgical interventions (3, 4). Electrical stimulation approaches to alter neurotransmission may be invasive or noninvasive. Some modalities include spinal cord stimulation (SCS), motor cortex stimulation, and deep brain stimulation (4–7). Targeted muscle reinnervation has also been shown to reduce phantom pain despite initially being developed for myoelectric prosthesis control (8).

By reducing pain intensity and frequency, successful treatment of PLP may improve a patient's overall function. This may be of particular importance for patients who have difficulty wearing and operating their prosthesis due to pain.

## Case description

A 52-year-old male with a prior injury to his left elbow subsequently requiring six surgical procedures developed complex regional pain syndrome (CRPS). His chronic pain score was 5/10 (Table 1). His condition was complicated by multiple contractures which limited function of his left upper extremity. With the goal of improving function, the patient underwent elective left transhumeral amputation with targeted muscle reinnervation. The patient planned to use a myoelectric prosthesis.

**Table 1:** Pain scores and functional measures at time points spanning pre-amputation to 2 years after spinal cord stimulator implantation.

Pain score or functional outcome	Pre-amputation	Post-amputation and pre-SCS	12 days post-SCS	2 years post-SCS
Pain score (10-point scale)	5	9	0	0
Sleep without interruption (hours)		3–4	8	
Sit without interruption (minutes)		0	30	
Read and retain information (minutes)		0	45	
Recovery after activity (hours)		Never	12	
Wear prosthesis (hours per day)		3	17	

Two months after amputation, the patient developed new left upper limb pain. He underwent multidisciplinary evaluation with physiatry and pain medicine, and history and physical exam were most consistent with the diagnosis of PLP. The patient also had some residual limb pain that was less severe than the PLP. Numerous pain management strategies were trialed including medications, therapies, and interventions. Medications trialed included gabapentin, pregabalin, nortriptyline, acetaminophen, NSAIDs, and prednisone taper. Therapies trialed included physical therapy and occupational therapy with modalities including mirror therapy, desensitization, massage, and passive range of motion exercises. Compression garments were used to help minimize edema and associated pressure on nerves in the residual limb. Interventions trialed included stellate ganglion nerve block. The patient did not have adequate pain relief or improvement in function from these pain management strategies.

PLP severity continued to increase and was complicated by involuntary muscle contractions. The patient's chronic pain score was 9/10 (Table 1). He had difficulty relaxing his residual limb when PLP was most intense. He had difficulty controlling his myoelectric prosthesis, and he was able to tolerate wearing his prosthesis only three hours per day.

**Figure 1:**
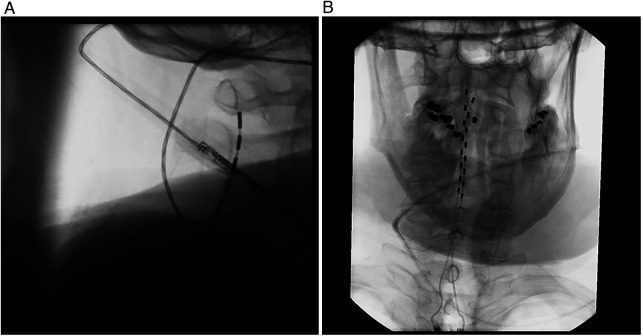
Fluoroscopic-guided cervical spinal cord stimulator placement. **(A)** Lateral view of the cervical spine showing the spinal cord stimulator leads within the epidural space to the level of C1-C2. **(B)** AP view of the spinal cord stimulator leads entering the epidural space at T1-T2 and traveling cephalad to the level of C1-C2.

**Figure 2:**
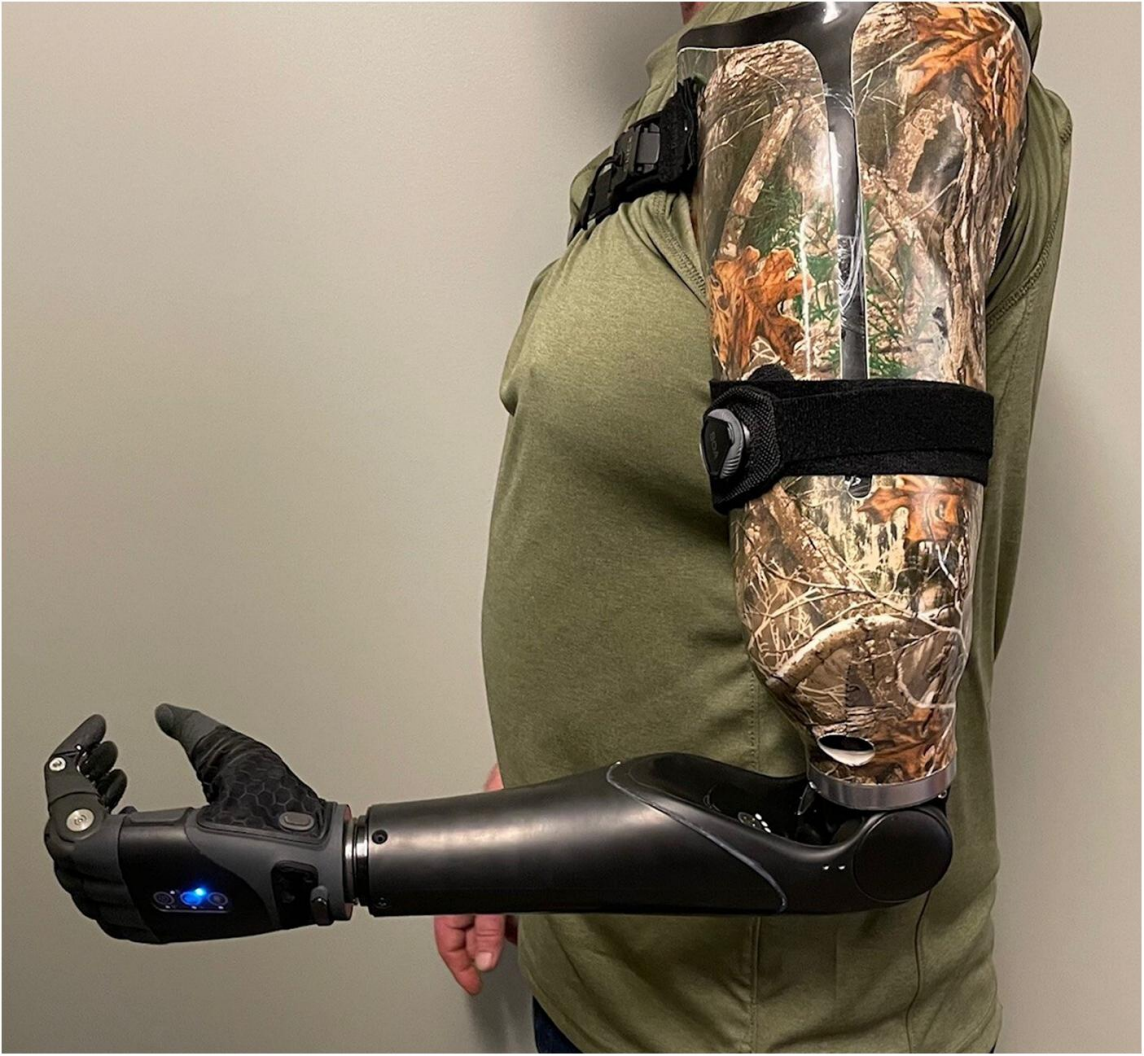
Patient wearing his myoelectric prosthesis.

The patient was re-evaluated by a pain medicine specialist and offered a cervical spinal cord stimulator for pain management. After a successful trial, a high frequency spinal cord stimulator (Nevro Corporation, Redwood City, CA) was implanted with two leads at the level of C1-C2 (Figure 1). The spinal cord stimulator was programmed to 10,000 Hz stimulation frequency. At 12 days post-procedure, the patient reported 70% improvement in his average pain intensity and a current pain score of 0/10. He also reported a subjective 70% improvement in overall function. Measured functional improvements included longer durations of sleeping without interruption, sitting without interruption, and reading and retaining information as well as shorter time to recover after activity (Table 1). The patient was able to tolerate wearing his prosthesis for up to 17 h per day. At six-month follow-up, the patient reported wearing his myoelectric prosthesis daily (Figure 2). At two-year follow-up, the patient reported a pain score of 0/10 (Table 1).

## Discussion

This case illustrates the profound functional impact PLP can have on patients with prosthetic devices. For the patient described in this case, high frequency SCS was an effective treatment for PLP after many other treatment options had been ineffective. The case is also an example of how SCS for the treatment of PLP can improve function for prosthetic device users. With improved pain control, patients with PLP may be able to tolerate wearing their prosthesis for more hours of the day. Improved pain control may also allow for more controlled muscle contraction and relaxation to better control the prosthesis; this is especially important for myoelectric prostheses.

The outcome of this case is consistent with previous literature describing SCS as an effective treatment for PLP (4). The predominant theories explaining the mechanism of SCS for the treatment of PLP are gate control theory (stimulation of A*β* fibers in the dorsal columns) and activation of descending inhibitory pathways, although other theories exist for SCS in the treatment of neuropathic pain more broadly (9, 10). For example, for the treatment of neuropathic pain and CRPS, SCS is thought to alter emotional processing by disrupting somatosensory-limbic connections (11). Further research is needed to delineate the specific pathophysiology of PLP and the mechanism of SCS in PLP. There is also evidence to support the idea that myoelectric prostheses may provide PLP relief, another reason that reducing barriers to prosthesis use is important (12).

In this case, high frequency SCS with 10,000 Hz was utilized, which was a mutual decision between the patient and the pain medicine specialist. One theory behind high frequency SCS for the treatment of pain is that stimulation may act directly on inhibitory interneurons in the dorsal horn (13). High frequency SCS is also a non-paresthesia-based programming (14). Given the minimal cerebrospinal fluid layer between the epidural space and the spinal cord in the cervical spine, over-stimulation can produce painful paresthesias in the upper extremities. The patient was able to avoid this complication with high frequency SCS.

While this case describes one treatment modality for PLP, other treatments may also improve function when effective. SCS is also used to treat CRPS, and it is possible that SCS reduced pain and improved function in this case in part due to the patient's history of pre-amputation CRPS (15).

Future research investigating treatments for PLP and other types of post-amputation pain should include functional outcomes and consider how pain impacts prosthetic limb use. While use of established functional assessment tools was limited in this patient's case, these tools can be helpful both for patient care and research in standardizing functional assessments. Measuring functional outcomes in patients undergoing treatment for PLP may inspire innovative treatment options that combine neuromodulation with prosthetic design (7).

## Data Availability

The original contributions presented in the study are included in the article, further inquiries can be directed to the corresponding author.
